# Supplementary material for: Anti-Inflammatory Effect of Simonsinol on Lipopolysaccharide Stimulated RAW264.7 Cells through Inactivation of NF-κB Signaling Pathway
Source: Molecules. 2020 Aug 6;25(16):3573. doi: 10.3390/molecules25163573 (PMC7463804; doi:10.3390/molecules25163573)
Supplement: Supplementary file 1 [file molecules-25-03573-s001.pdf]

Elmt	Val.	Min	Max	Elmt	Val.	Min	Max	Use Adduct
H	1	0	300	O	2	0	50	H
C	4	0	150					
N	3	0	0					

Error Margin (ppm):	50	DBE Range:	-2.0 - 50.0	Electron Ions:	odd
HC Ratio:	0.0 - 5.0	Apply N Rule:	yes	Use MSn Info:	no
Max Isotopes:	all	Isotope RI (%):	1.00	Isotope Res:	10000
MSn Iso RI (%):	75.00	MSn Logic Mode:	AND	Max Results:	10

Event#: 4 MS(E-) Ret. Time : 26.392 Scan# : 3029

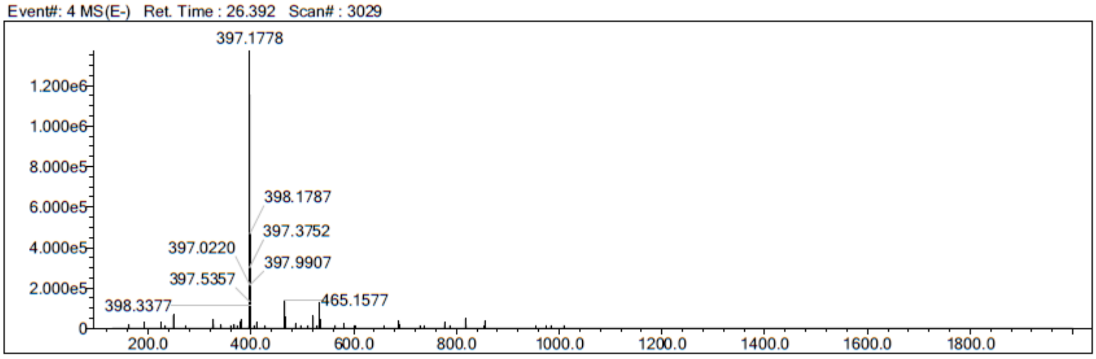

Measured region for 397.1778 m/z

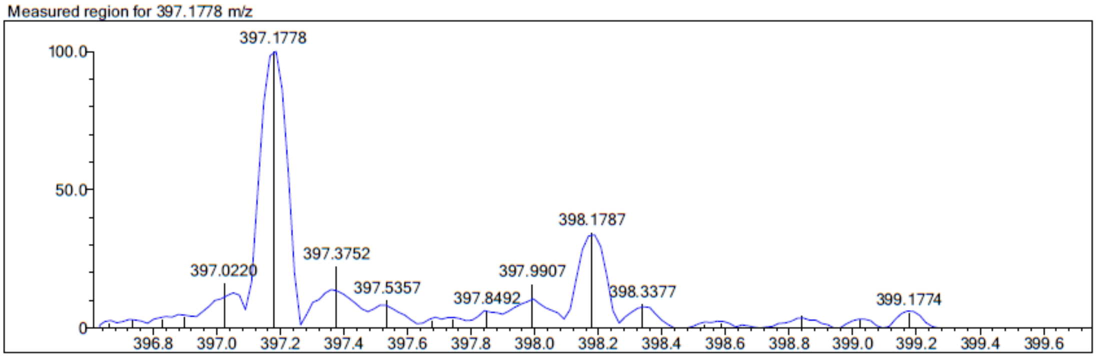

C27 H26 O3 [M-H]- : Predicted region for 397.1809 m/z

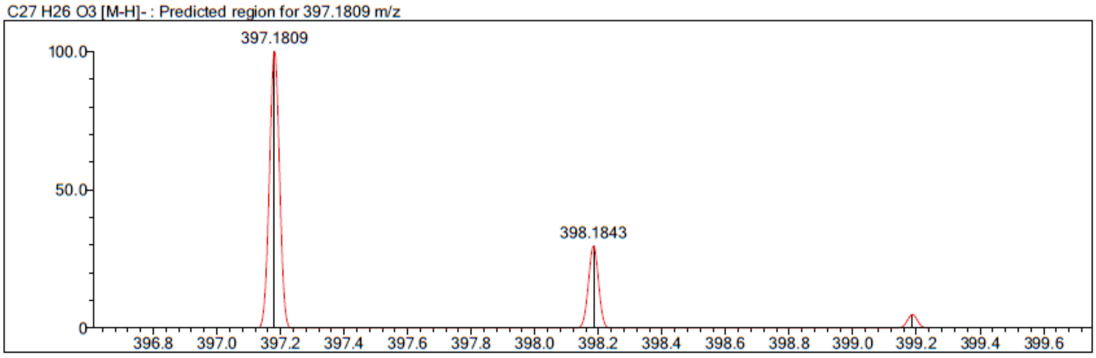

Rank	Score	Formula (M)	Ion	Meas. m/z	Pred. m/z	Df. (mDa)	Df. (ppm)	Iso	DBE
1	47.09	C27 H26 O3	[M-H]-	397.1778	397.1809	-3.1	-7.81	76.08	15.0

Fig. S1: HR-ESI-MS spectrum of **1**

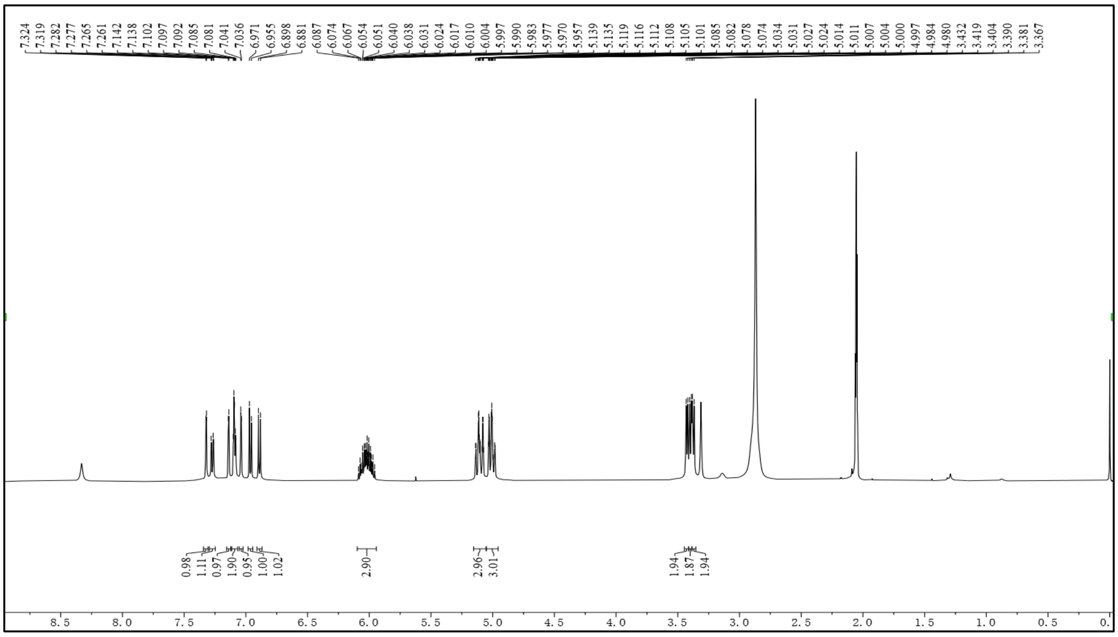

Fig. S2: ^{1}H-NMR spectrum of **1** (500 MHz, acetone-*d6*)

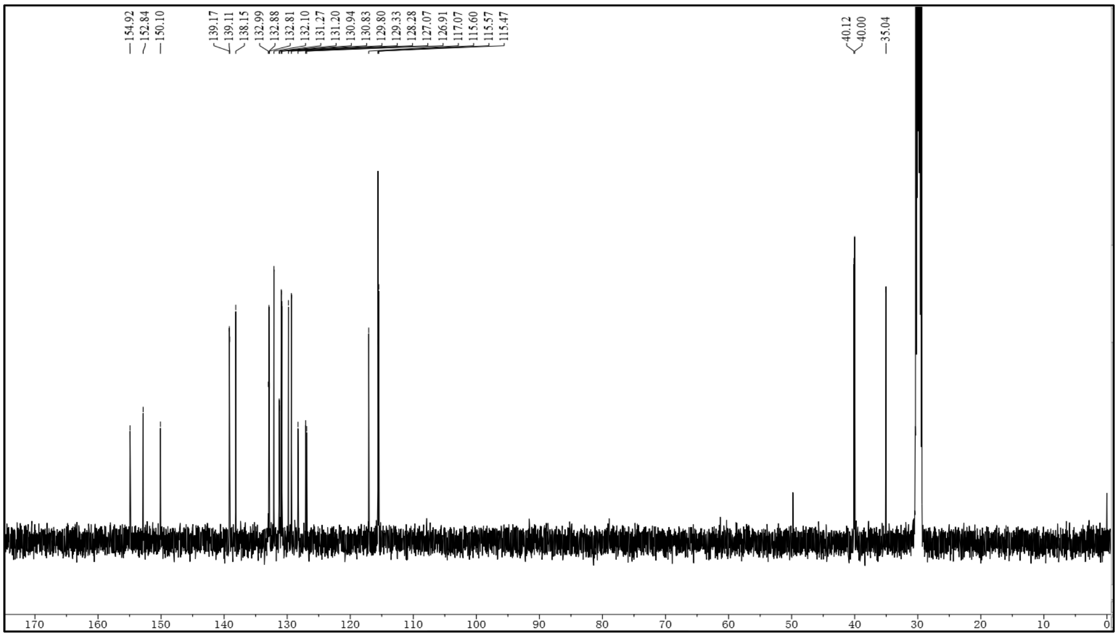

Fig. S3: ^{13}C-NMR spectrum of **1** (125 MHz, acetone-*d6*)

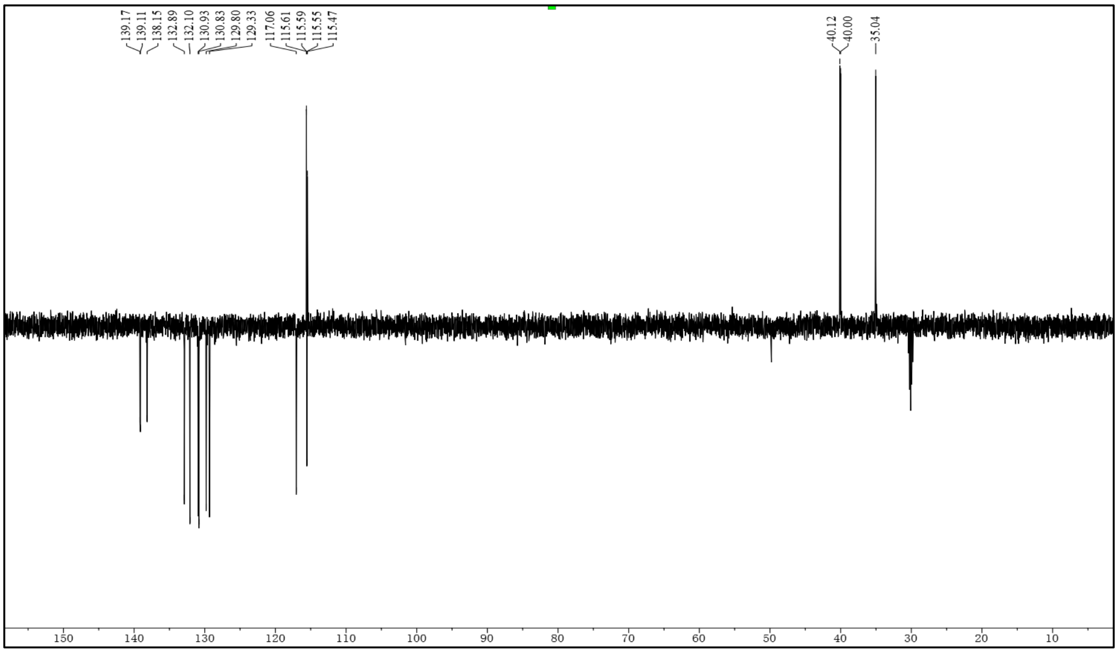

Fig. S4: Dept135 spectrum of **1** (125 MHz, acetone-*d6*)

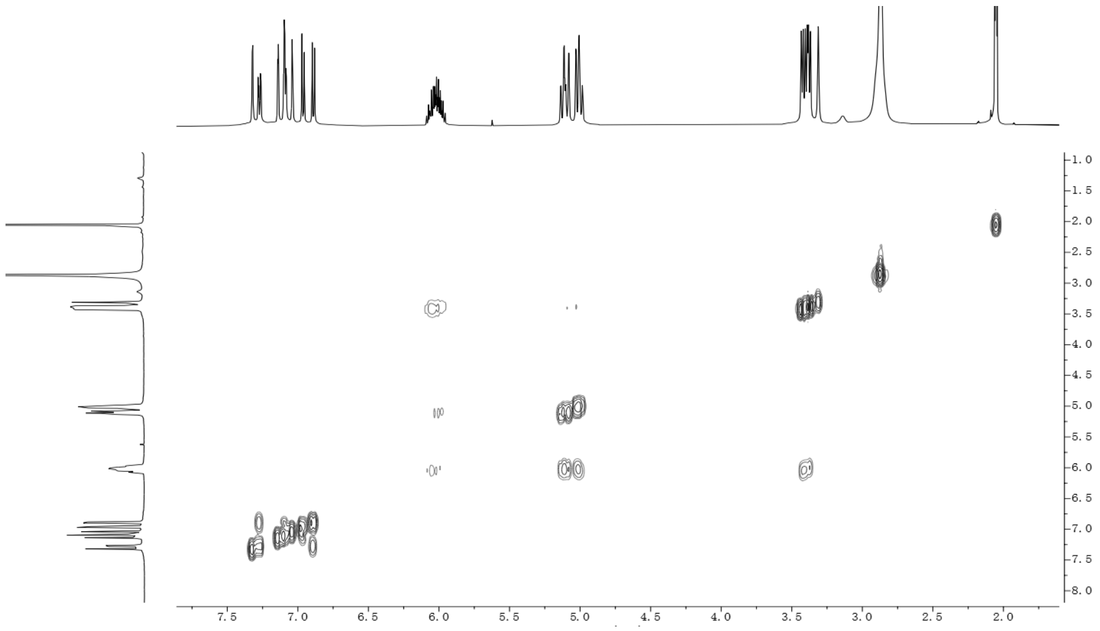

Fig. S5: ^{1}H-^{1}H COSY spectrum of **1**

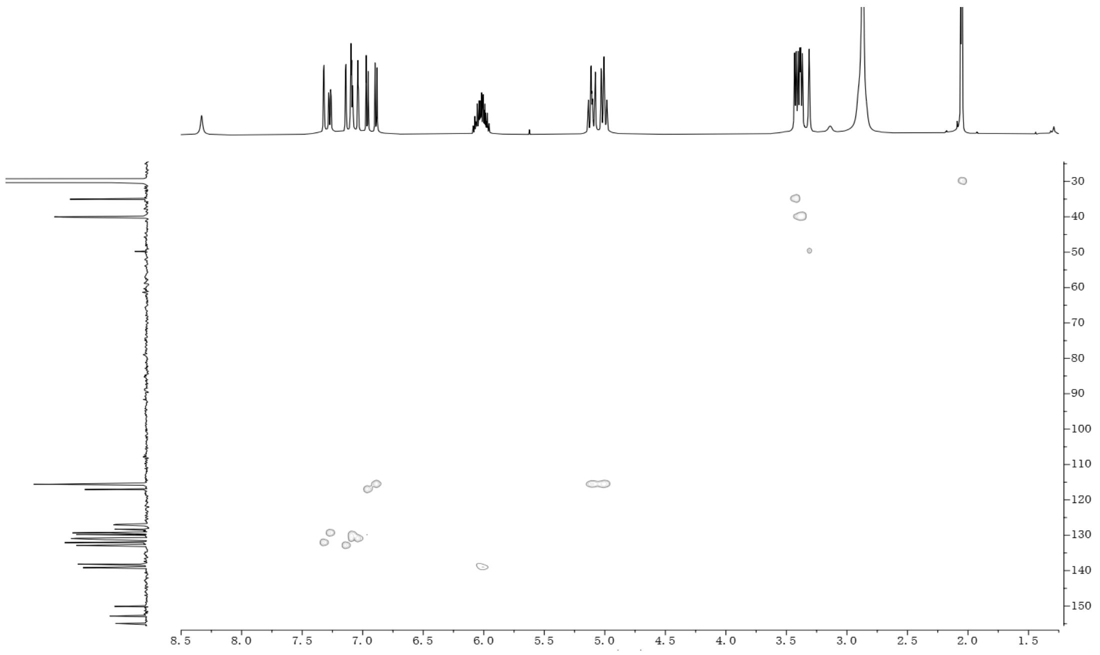

Fig. S6: HSQC spectrum of **1**

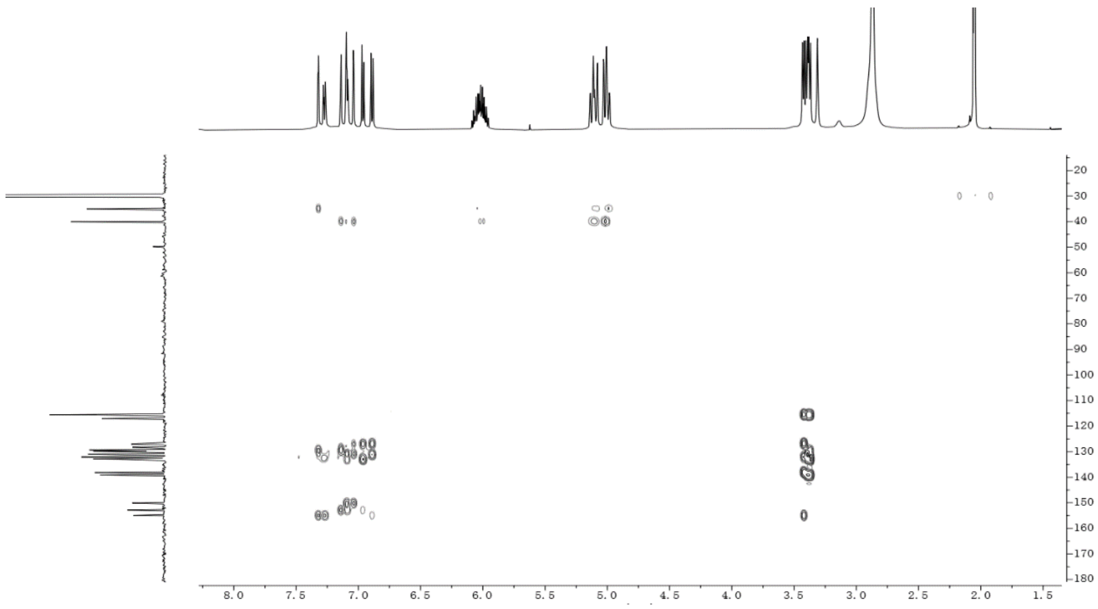

Fig. S7: HMBC spectrum of **1**

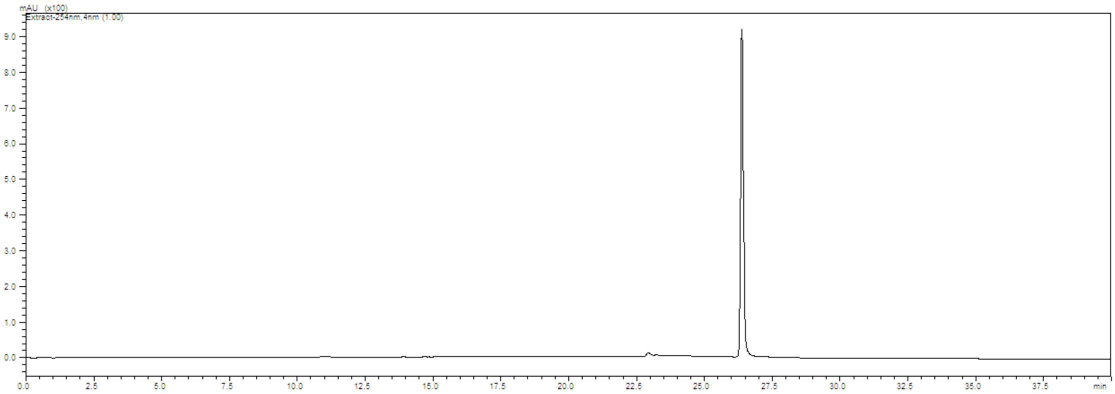

Fig. S8: HPLC spectrum of **1.** Compound 1 was performed on a polans 5 C-18-A HPLC column (150 × 2.0 mm) with a gradient elution of acetonitrile (A) and water (B) (0.-18 min, 5%-55%A, 18-25 min, 55%-90% A, 25-30 min, 90%-90% A, 30-40 min, 90%-100%A) at flow rate of 0.3 ml/min.
